# Therapeutical and Pharmaceutical Notes

**Published:** 1899-06

**Authors:** W. J. Martin

**Affiliations:** Kankakee, Ill.


### THERAPEUTICAL AND PHARMACEUTICAL NOTES.

Under the Direction of W. J. Martin, M.D.C.,
KANKAKEE, ILL.

Solution for Burns. No. 1: ℞. Acid picric, 10 parts; acid citric, 20 parts; water up to 100 parts. Mix. This solution is best applied with a pledget of cotton-wool or a soft sponge, and the burned surface, where practicable, should afterward be well covered with the same material. The dressing may be reapplied after the lapse of two or three days.

Solution No. 2: ℞. Acid picric, ʒiv.; alcohol, ℥iv.; water, distilled, ℥viij. Mix and use as No. 1.

The Use of Rectal Injections of Salt Water in Place of Injections of Artificial Serum into the Veins. The *Journal de Médicine* of Paris tells us that Pauchet has used salt water in the proportion of 8 per 1000 in hemorrhage after surgical operations, and in parturition by means of rectal injections, with very good results.

It is well known, of course, that these injections can be given intravenously in case of great hemorrhage accompanied by shock, and that they frequently do a great deal of good. So, too, subcutaneous injections of artificial serum are often exceedingly valuable. Pauchet claims, however, that the use of saline solution by the bowel is followed by good results, and has none of the disadvantages of either the intravenous or hypodermatic injections. The patient receives a rectal injection of a pint of hot salt solution, and this may be repeated, if the collapse is great or the hemorrhage has been great, two or three times during the course of the day. He has known of cases where as much as three quarts have been absorbed in the course of twenty-four hours. Should the bowel become intolerant, then other methods have to be resorted to, but Pauchet asserts that the pulse improves, the urine becomes abundant, the mouth becomes moist, and that this treatment also seems to diminish the vomiting following the use of an anæsthetic. A similar recommendation to that of Pauchet is made by Fieux in regard to post-partum hemorrhage, he also claiming that such injections are an exceedingly valuable method of treatment—*Therapeutic Gazette.*

[We have been using during the past year rectal injections of hot salt solutions in the treatment of mares after difficult parturition; in horses where, from an injury, a large amount of blood had been lost; in the more serious cases of azoturia, more especially in heavy draught animals, and find that its therapeutical effects are most beneficial.—W. J. M.]

DEODORIZED PETROLEUM OIL. One ounce of C. P. nitric acid added to one pint of kerosene oil will effectually remove the petroleum odor of the oil.

ACOIN. A new cocaine succedaneum, said (*Therap. Monat.*) to be much less poisonous than cocaine, and to act longer and in weaker solution. Concentrated solutions should not be used, for acoin has a corrosive action when applied in a concentrated form. For subcutaneous injection a solution composed of one part of acoin, eight parts of sodium chloride, and a thousand parts of distilled water should be used. This solution should be protected from light.

INCOMPATIBILITY OF IODIDE OF POTASSIUM AND MERCURIAL OINTMENT. It has lately been discovered by a French chemist, M. Mausier, that a mixture of iodide of potassium and mercurial ointment are incompatible. On mixing these two substances together by trituration there are formed two irritating salts, the mercurous and mercuric iodides.

COCAINE HYDROCHLORATE INCOMPATIBLE WITH CHLOROFORM WATER; NEW TEST FOR COCAINE. Cocaine hydrochlorate should never be added to saturated chloroform water, writes M. Mausier, in the *Centre Med. et. Pharmaceutique.* Chloroform being much less soluble in water containing the cocaine salt than in pure water, the result of the addition of cocaine to the chloroform water is the precipitation of chloroform in very minute drops, which find their way to the bottom of the container. When it becomes necessary or desirable to use the combination the chloroform water should not be of the saturated strength; or, if one must prescribe the saturated water, he should add either 1 per cent. of citric acid or 5 per cent. of alcohol to the mixture, both of which augment the solubility of the chloroform water. Saturated chloroform water, on account of the reaction, may serve as a reagent in the identification of cocaine. Of over a dozen of the alkaloids in common use tested the salt of cocaine alone caused the precipitation of chloroform from its saturated solution.—*Bull. Pharmacy.*

Alcohol as an Antidote for Carbolic-acid Poisoning. In the *New York Medical Journal*, January 14, 1899, Dr. A. M. Phelyn tells us that Dr. Seneca D. Powell, of New York, has for a long time used in his clinic at the Post-graduate Hospital an antidote that all have come to recognize as a specific. He alludes to alcohol, and it is not an unusual occurrence to see Dr. Powell, in the presence of the class, catch in his open hands a quantity of pure carbolic acid poured into them by a nurse from a bottle. In a few moments the doctor puts his hands into a basin of pure alcohol, and no escharotic effect is observed whatever from the action of the carbolic acid upon the skin. Dr. Phelyn says he was somewhat surprised when he first saw this experiment, but when he recognized the result he was convinced of the scientific fact. At the present time he is flushing out abscess cavities with pure carbolic acid and washing them out a few moments later with pure alcohol. In empyema Dr. Powell, after making a large opening in the chest-wall, washes out the cavity with a 10 per cent. solution of carbolic acid, after which pure alcohol is used, and no bad effect has thus far been observed from this treatment. The cavity of the pleura is rendered aseptic. From personal observation and demonstration in the use of pure carbolic acid followed by the use of alcohol, Dr. Phelyn says he can state to the profession positively that we have in alcohol an absolutely safe and sure specific against the escharotic action of pure carbolic acid; and he believes that this fact should be given wide publication to the profession and even to the laity, because in cases of carbolic-acid poisoning with homicidal intent, if, immediately after the administration of the poison, alcohol was thrown into the stomach of the individual, the poisonous effect of carbolic acid would at once be neutralized. —*Therap. Gazette.*

Medicinal Value of Sarsaparilla. It has been recently stated by a prominent lecturer on pharmacognosy that sarsaparilla as ordinarily found in the drug trade contains no more medicinal virtues than so much chips.

Germol. Germol is the name of an English disinfectant similar to creolin. It is a limpid, dark-brown liquid, smelling strongly of cresol. It is neutral, and added to water in moderate proportion yields a white emulsion. The manufacturers hint that it is a distillate of peat, and consists of cresylic and carbolic acids and their homologues, mixed with naphthalin and its homologues. "Chemically," they say, "it is one hundred disinfectant." Just what is the composition we are not prepared to say. It seems to be purified cresol rendered water-miscible with soap.—*Western Druggist.*

SANTONIN FOR WORMS IN PIGS. It is reported (*Drug Circular*) that farmers in Iowa have taken to feeding santonin to their pigs as a vermifuge, and that the good results are most surprising. The drug is administered by stirring one ounce of it into the swill intended as one meal for thirty-five pigs.

VANADIN. This is a solution of a vanadium salt and chlorate of sodium, and has lately been proposed as a specific for pulmonary tuberculosis in man. The dose is from six to thirty drops.

CARBO-SAPOL. Dr. George T. Beatron (*British Medical Journal*) very highly recommends a compound named by its originator, J. McMillan, "carbo-sapol." It is a mixture of 50 per cent. of Calvert's No. 5 carbolic acid, 25 per cent.; Tennant's yellow soap, and 25 per cent. soft black soap. The ingredients are heated together in a water-bath until a clear solution is obtained; the mixture is then strained through lint. It is perfectly miscible with water, forming an oleaginous solution, which yields an abundant lather when the hands are washed in it, leaving them, when dry, soft and glossy. It is less irritating than the ordinary carbolic lotion, a 1 per cent. solution being strong enough for use of the hand or the skin of a patient. During operation carbo-sapol is of great service both for the hands and sponges. Corrosive sublimate solution forms with blood a compound which stains the skin and nails in a disagreeable way, and sponges with blood in them are also permanently discolored. A preliminary washing in a 1 per cent. solution of carbo-sapol gets rid of the objectionable blood, after which a 1 : 2000 corrosive lotion may be used to remove the slipperiness from the hands when handling the instruments.

TANALBIN. This drug is an albuminate of tannin, exsiccated, and is said to be a specific remedy in the diarrhœas and dysenteries so commonly met with in domestic animals. It is a light brown powder, insoluble in water, tasteless and odorless. Being insoluble in the gastric juice of the stomach, it causes no disturbance in that organ. It is slowly soluble in the intestinal fluids, and has a firm, astringent action on the enteric mucosa of both the large and small intestines. It is said to have no effect on the mucosa of a healthy intestine, being there entirely innocuous.

Tanalbin has been used quite extensively by European veterinarians and to a limited extent by American veterinarians. The reports concerning the clinical results following the use of this drug are highly commendable to it. The dose of tanalbin for colts and calves two to eight weeks old is forty-five grains, given three times a day mixed with syrup or gruel. It may also be given, in combination with opium, in pill form. Horses and cattle take from one and one-half to two and one-half drachms three times daily.

From Germany comes a suggestion for the manufacture of an article quite equal to tanalbin, which can be produced at much less cost. The directions to accomplish this are about as follows: A mixture is made of albumin solution, 10 per cent., 10 parts; tannin, 10 per cent., 6.5 parts, from which a precipitate is collected. After washing well, pressing out, and drying at 86° F. it may be powdered, sifted through a fine sieve, and spread out thinly, to be heated for about six hours at a temperature of 240° F. This product is claimed to do all that the previous new product has done.—*Ephemeris.*

FATAL ACTION OF FORMALDEHYDE. A correspondent of the *American Druggist* relates how, for the purpose of killing fleas, a large dog was sponged with a solution of five teaspoonfuls of formalin (40 per cent.) in three-fourths of a pail of water. Soon after the application the animal was found to be paralyzed, and died later on.

METHYLENE-BLUE. This drug belongs to the aniline group of derivatives, and has been of late quite extensively used in human practice. "It is useful in epithelial nephritis by increasing diuresis and causing the albumin entirely to disappear. In cystitis both internal administration and that by injection of a dilute aqueous solution resulted in marked benefit. It is an excellent germicide and analgesic, and prevents fermentation."—*Bull. Gen. de Therap.*

In the treatment of continued malarial fever in man a combination of methylene-blue and quinine gave decided beneficial results when both drugs used separately had failed to give relief. The dose of methylene-blue for an adult person is from ten to twelve grains. For veterinary purposes the dose can be easily graded from this, according to the species of animal to which administered.

Adulteration of Creosote. Upon examination by the committee on adulteration of the New York Pharmaceutical Association it was found that 50 per cent. of the article sold in the open market as creosote consisted of carbolic acid diluted with more or less water, alcohol, and glycerin.

Holocaine. A new synthetic anæsthetic, used principally in eye surgery, is very rapid in its action, but superficial in its effects, and is therefore inferior to cocaine for the deeper operations on the eye. It may be used in solutions containing 1 to 2 per cent. of the drug. Solutions of this strength are found to possess decided germicidal properties, and are therefore of value in treating all traumatic affections of the eye.

Hypodermic Eucaine Solution. Dr. Braun uses the following solution of eucaine for hypodermatic purposes as probably the least immediately painful, the safest, and most efficient yet advised: Eucaine B., 1 grain; sodium chloride, 8 grains; distilled water, 2 fluidounces. This keeps well, and can be boiled an indefinite number of times without deteriorating. It should be about blood-heat when injected. It is osmotically indifferent, causes no irritation, and induces an anæsthesia which lasts from ten minutes to an hour, according to the quantity injected.—*Western Druggist*

Cocaine Solution. It has lately been proved that aqueous solutions of cocaine do not have to be sterilized by heat for their preservation if one centigramme of salicylic acid is added to each ten grammes of liquid. A similar preparation is recommended by the *British Pharmacopœia*, composed of: Hydrochlorate of cocaine, 15 grains; distilled water, 2 ounces; salicylic acid, 7 grains.—*Therapeutic Gazette.*

Phenosalyl. This compound has been found to be an efficient agent in the treatment of erysipelas and other phlegmonous diseases of the skin where a thoroughly reliable antiseptic is desired. Its composition is as follows: Acid phenol, 80 parts; salicylic acid, 10 parts; lactic acid, 10 parts; menthol, 1 part. Mix.

